# Identification of FVIII gene mutations in patients with hemophilia A using new combinatorial sequencing by hybridization

**DOI:** 10.4103/0971-6866.44106

**Published:** 2008

**Authors:** M. Chetta, A. Drmanac, R. Santacroce, E. Grandone, S. Surrey, P. Fortina, M. Margaglione

**Affiliations:** 1 Genetica Medica, Dipartimento di Science Biomediche, Universita' degli Studi di Foggia, Foggia, Italy; 2 Callida Genomics, Sunnyvale, CA; 3 Department of Medicine, Thomas Jefferson University, Jefferson Medical College, Philadelphia, PA; 4 Unità di Emostasi e Trombosi, IRCSS “Casa Sollievo della Sofferenza”, San Giovanni Rotondo, Fg, Italy

**Keywords:** Combinatorial sequencing-by-hybridization, FVIII gene, hemophilia A

## Abstract

**BACKGROUND:**

Standard methods of mutation detection are time consuming in Hemophilia A (HA) rendering their application unavailable in some analysis such as prenatal diagnosis.

**OBJECTIVES:**

To evaluate the feasibility of combinatorial sequencing-by-hybridization (cSBH) as an alternative and reliable tool for mutation detection in FVIII gene.

**PATIENTS/METHODS:**

We have applied a new method of cSBH that uses two different colors for detection of multiple point mutations in the FVIII gene. The 26 exons encompassing the HA gene were analyzed in 7 newly diagnosed Italian patients and in 19 previously characterized individuals with FVIII deficiency.

**RESULTS:**

Data show that, when solution-phase TAMRA and QUASAR labeled 5-mer oligonucleotide sets mixed with unlabeled target PCR templates are co-hybridized in the presence of DNA ligase to universal 6-mer oligonucleotide probe-based arrays, a number of mutations can be successfully detected. The technique was reliable also in identifying a mutant FVIII allele in an obligate heterozygote. A novel missense mutation (Leu1843Thr) in exon 16 and three novel neutral polymorphisms are presented with an updated protocol for 2-color cSBH.

**CONCLUSIONS:**

cSBH is a reliable tool for mutation detection in FVIII gene and may represent a complementary method for the genetic screening of HA patients.

## Introduction

Hemophilia A (HA) is a common inherited recessive X-linked disorder of blood clotting caused by deficiency of factor VIII in the coagulation cascade and affects approximately 1 in 5,000 males world-wide.[1,2] The FVIII gene, comprises 26 exons ranging from 69 bp (exon 5) to 3.1 kb (exon 14) in size, spans 186 kb of genomic DNA and produces a 9030 nt mRNA. According to the UK Hemophilia Centre Doctors' Organisation (UKHCDO) Hemophilia Genetics Laboratory Network, the severity of HA in the pedigree should be determined first as this will influence the diagnostic strategy to be employed. Severe Hemophiliacs should be screened for the intron 22 inversion mutation followed by the intron 1 inversion mutation. This approach identifies the underlying mutation in 45-50% of severe HA patients.[3,4] The remaining severe HA pedigrees should then be analyzed further either by full mutation or linkage analysis. Mutations have been found in nearly all 26 exons of the factor VIII gene, over 400 mutations have been identified[5,6] and de novo mutations represent approximately 30% of all cases.[7] The most common detection methods include DNA sequence analysis which requires numerous reactions and individual analysis of each exon or alternative screening methods such as single-stranded conformation polymorphism (SSCP),[8] denaturing gradient gel electrophoresis (DGGE)[8] and denaturing high performance liquid chromatography (dHPLC).[9] We applied the new combinatorial sequencing-by-hybridization (cSBH) as an alternative method to the traditional Sanger dideoxy chain termination approach.[10,11] Previous works have shown that cSBH is an efficient rapid and alternative method for mutation detection.[12,13]

We increased the quality of results with a new cSBH method that use two different colors (TAMRA and QUASAR). The platform is an indirect method which uses standard chemistry of base-specific hybridization of complementary nucleic acids to indirectly assemble the order of bases in a target DNA. Short oligonucleotide probes are arrayed in the form of high-density arrays of universal sequence and hybridized to sample DNA molecules. The resulting hybridization pattern is used to generate the target sequence using computer algorithms.

We report development of a strategy to implement 2-color cSBH to screen a range of mutations within the FVIII gene.

## Materials and Methods

### Sample

After approval of the local ethics committee, the study was carried out according to the Principles of the Declaration of Helsinki. After informed consent the genomic DNA was isolated from clinically diagnosed individuals with HA, regularly investigated for the detection of the causative mutation within the F8 gene. Samples were labelled using an internal lab code. We selected samples that presented a point mutation at different position in the F8 gene. We selected samples 1 and 20, which present a nonsense mutation; samples 73 and 95, which present a 2 bp deletion; samples 2, 6, 37, and 89, which present a single bp deletion; and a series of randomly selected samples in which a missense mutation was identified. Samples 1 and 20 and 2, 6, and 37 carry the same mutation and were used to verify the quality of results in the analysis. In addition, we randomly selected 7 HA male patients, who were previously excluded to carry the inversion of the intron 1 or of the intron 22 and in whom no gene mutation was detected by a first FVIII gene screening, using common strategies (SSCP, DGGE, or dHPLC). All the patients involved in this study are regularly followed at one of 50 hemophilia treatment centers spread all over the country and belonged to the Italian Association of Hemophilia Centers (AICE; *URL: http://www.aiceonline.it*), an organization which was founded with the aim to organize clinical and experimental national activities in inherited bleeding disorders. Patients were considered to have HA according to the international consensus of the 2001 International Society on Thrombosis and Haemostasis (ISTH) Factor VIII and Factor IX Subcommittee.[9] All known HA patients are registered and followed at one of these centers. For each patient clinical and laboratory data (including FVIII clotting activity and inhibitor) were recorded. DNA was extracted from 5 to 10 ml of EDTA treated peripheral blood leukocytes with in standard salting out method. Numbering of mutations and polymorphisms was in agreement with genomic DNA sequence in Hamsters database mutations (http://europium.csc.mrc.ac.uk/WebPages/Main/main.htm). Patient DNA was amplified for exons spanning the FVIII gene and encompassing each different disease-causing mutation.

### Primer design

Primers for multiplex and nested PCR were obtained from MWG Deutschland, (HPLC purified) and designed using Primer 3 software (www.genome.wi.mit.edu/cgibin/primer/primer3_www.cgi) to span the entire HA gene coding regions with intron-exon boundary coverage.

### DNA amplification

The amplification of target for cSBH was performed in two consecutive steps [Table 1]. The first required three different multiplex PCR reactions to amplify all exons while the second step, a nested-asymmetric PCR, generated single-stranded templates corresponding to sense and antisense strands. The multiplex PCR was performed in 25 µ l containing 100 ng of genomic DNA, 33 pmol of each primer suspended in three different mixes (Mix 1, 2, and 3 with primers for exons 1-9, 10-13 and 15-25, and 14 and 26, respectively). Reactions were performed for 40 cycles with an initial denaturation at 96°C for 15 min. The protocols to amplify Mix 1 required an initial denaturation at 96°C for 15 min, followed by 20 cycles of denaturation (96°C, 30 sec), annealing (52°C for 30 sec), and extension (72°C, 50 sec); while for Mix 2, 20 cycles of denaturation (96°C, 30 sec), annealing (55°C for 30 sec) and extension (72°C, 50 sec) with a final extension at 72°C for 7 min were required. In Mix 3 the annealing temperatures are 55°C and 58°C for exons 14 and 26, respectively. This separation is required to optimize the amplification conditions for all exons.

**Table 1:** Sequence and position of primers used for multiplex and nested PCR reactions

Exon	Multiplex PCR Sequence primers	Position*	Exon	Nested PCR Sequence primers	Position*	PCR Product
Ex1_F	gttcttcctgtggctgcttc	-55/-35°	Ex1_Fn	gctgcttcccactgataaaaa	-43/-22 °	387 bp
Ex1_R	agcagacttacatccccacaa	353-373	Ex1_Rn	ccgatcagaccctacaggac	325-344	
Ex2_F	ttcaaatttgcctccttgct	23012-23032	Ex2_Fn	cagctgctttttgaagtgtcc	23068-23089	217 bp
Ex2_R	tttggcagctgcacttttta	23328-23348	Ex2_Rn	cccaatttcataaatagcattcaa	23261-23284	
Ex3_F	tttggaataacaggttttctgga	25562-25585	Ex3_Fn	ctttggcggacatctcattc	25602-25623	180 bp
Ex3_R	gccaccattacaaagcacac	25808-25828	Ex3_Rn	tgacaggacaataggagggta	25661-25682	
Ex4_F	cagtggatatagaaaggacaatttta	29534-29560	Ex4_F	cagtggatatagaaaggacaatttta	29534-29560	281 bp
Ex4_R	ttcagttgtttgtacttctctgctt	29823-29848	Ex4_R	ttcagttgtttgtacttctctgctt	29823-29848	
Ex5_F	ttgttcttactgtcaagtaactgatga	35313-35340	Ex5_Fn	ttgttcttactgtcaagtaactgatga	35313-35340	168 bp
Ex5_R	tgatctttctataatcacctcctctt	35544-35570	Ex5_Rn	tgatctttctataatcacctcctctt	35544-35570	
Ex6_F	gagcagggaaggagaaagg	37840-37859	Ex6_Fn	gcggtcattcatgagacaca	37863-37883	215 bp
Ex6_R	ccgagctgtttgtgaactga	38101-38121	Ex6_Rn	ttggaagaccctgaggattg	38058-38078	
Ex7_F	tttgtccattctgtcctagcaa	53089-53111	Ex7_Fn	tcatagccataggtgtcttattcc	53138-53162	350 bp
Ex7_R	aatgtccccttcagcaacac	53481-53501	Ex7_Rn	gcaacacactatattcctgtacattg	53462-53488	
Ex8_F	tagcctgcagaaacatgagc	55919-55939	Ex8_Fn	tgtttggtttgtctgactcca	56015-56036	322 bp
Ex8_R	tggcttcaggatttgttggt	56365-56385	Ex8_Rn	tggggaagagagagtaccaat	56316-56337	
Ex9_F	cactccttgccttgattgaa	56481-56501	Ex9_Fn	tttttcttcccaacctctcatc	56543-56565	260 bp
Ex9_R	ccattggagacaaggctgaa	56833-56853	Ex9_Rn	tccagactttttcttcttacctga	56779-56803	
Ex10_F	gaggccacttttatttatctgga	61462-61485	Ex10_Fn	gaccacagttttcttgttgatcc	61490-61511	192 bp
Ex10_R	ggaccaacataattttagttgttattg	61711-61738	Ex10_Rn	aaaaagagctataaatcgagggaat	61656-61680	
Ex 11_F	gaacccttgcaacaacaaca	65470-65490	Ex11_Fn	atggttttgcttgtgggtag	65533-65553	260 bp
Ex11_R	ggggacatacactgagaatgaa	65808-65830	Ex11_Rn	agcacttggaaaggcaagaa	65783-65803	
Ex12_F	tgctagctcctacctgacaaca	68617-68638	Ex12_Fn	gcatttctttacccctttcaa	68645-68666	230 bp
Ex12_R	cattcattatctggacatcactttg	68890-68915	Ex12_Rn	ctttattcaccacccactgga	68854-68875	
Ex13_F	tcatgacaatcacaatccaaaa	74746-74767	Ex13_Fn	cttttccccattgtttttgc	74795-74815	273 bp
Ex13_R	gagcatacgaatggctagtgaa	75068-75083	Ex13_Rn	gcattcacagctgttggtaca	75047-75067	
			Ex14-1_Fn	gagtcttattcttcctcatctcca	91023-91047	1022 bp
			Ex14-1_Rn	gggccatcaatgtgagtctt	92025-92044	
Ex14_F	ctgggaatgggagagaacct	90965-90985	Ex14-2_Fn	caaaacttccaataattcagcaa	91994-92017	1176 bp
Ex14_R	ttgttggtgtcatcatctgg	94206-94226	Ex14-2_Rn	acctttgcaatgggtaatgg	93150-93170	
			Ex14-3_Fn	cgagcaccctcacacagata	93037-93057	1156 bp
			Ex14-3_Rn	cacaagagcagagcaaagga	94173-94193	
Ex15_F	gcttggcaagagtatttcaagg	116010-116032	Ex15_Fn	ggcacctaggaaaatgagga	116067-116087	269 bp
Ex15_R	aaaatttccaaaagtgggaataca	116371-116393	Ex15_Rn	ttttcttgtaattccactgtcctt	116312-116336	
Ex16_F	aaagacaggtatttcttttagggatg	117604-117630	Ex16_Fn	gatcctagaagattattcttgcttttt	117648-117676	300 bp
Ex16_R	ttgcacgtaggataaatatcaaaa	117981-118003	Ex16_Rn	ccaaaaagtggtcagcacaa	117929-117949	
Ex17_F	tctggttcataggtgagagagc	118144-118166	Ex17_Fn	gattgatgtcttccctcccta	118178-118199	301 bp
Ex17_R	ccctggatcaagtctcatttg	118484- 118505	Ex17_Rn	agtgcaatctgcatttcacag	118458-118479	
Ex18_F	tggtggagtggagagaaagaa	118570-118591	Ex18_Fn	ggcttctctgtgtccttctcc	118613-118634	229 bp
Ex18_R	gtgttcccagtgcctagacc	118880-118900	Ex18_Rn	aaagagcacaaacaagctcatac	118819-118842	
Ex19_F	ttcgcataaaccaatgtatctca	120481-120504	Ex19_Fn	aatttctgttcctgttggttttt	120529-10551	169 bp
Ex19_R	gcaaccattccagaaaggaa	120733-120753	Ex19_Rn	acctctgcccacattgctac	120678-120698	
Ex20_F	ttgacgttctcccattttca	121194-121213	Ex20_Fn	gtgttccacccgtttcattt	121259-121279	134 bp
Ex20_R	gaagcatggagatggattcatta	121430-121453	Ex20_Rn	atcagcccaggttcttgga	121374-121393	
Ex21_F	tgtctaggactaacccagctgaa	122719-122742	Ex21_Fn	tctgtttctttacttgggcaaa	122750-122761	146 bp
Ex21_R	tttgagcttgcaagaggaataa	122914-122936	Ex21_Rn	tgtgatacatttcccatcattg	122874-122896	
Ex22_F	tgatggacacacctgtagcaa	126381-126402	Ex22_Fn	tttcaggaggtagcacatacattt	126431-126455	250 bp
Ex22_R	ccaatatctgaaatctgccaaa	126695-126716	Ex22_Rn	caggcattccctttaaatgac	126659-126680	
Ex23_F	gttgagaaacgcacaaagca	159393-159413	Ex23_Fn	ccatgtatttgtgctaatctctcc	159467-159491	195 bp
Ex23_R	catggttgagggaagaagga	159664-159684	Ex23_Rn	tgggatgacttggcacttac	159642-159661	
Ex24_F	tcagtggaagctgctcagta	160784-160803	Ex24_Fn	actgaggctgaagcatgtcc	160806-160826	250 bp
Ex24_R	aggccttccccgattttt	161059-161102	Ex24_Rn	cccaaccactgctctgagtc	161036-161056	
Ex25_F	tgagaagtgctgtggtatggtt	162023-162055	Ex25_Fn	agggatttgggaatttctgg	162059-162067	336 bp
Ex25_R	tgttaagctctaggagaggtggt	162336-162359	Ex25_Rn	tctttccaaggagaccagctt	162294-162315	
			Ex26-1_Fn	ctgtcagacaaccaataaatgct	184933-184956	999bp
Ex26_F	cagtgaccattgtcctgtca	184919-184939	Ex26-1_Rn	ttgcatcctcctgacttattttt	185909-185932	
Ex26_R	tcagtgttcacatttttatttcca	+11/+36#	Ex26-2_Fn	caggaggatgcaattgttga	185919-185938	1048 bp
			Ex26-2_Rn	tccagtgtaaaaacacaattctcaa	+33/+56#	

* numbering according to the GeneBank NG_005114 sequence.

° nucleotides upstream the start of the NG_005114 sequence.

# nucleotides downstream the NG_005114 sequence.

The nested PCR amplification for all exons was performed in 100 µ l reactions containing a 1:4 dilution of multiplex PCR reaction (25 µ l multiplex reaction in 75 µ l of ddH_2_0), 30 pmol of forward primer and 3 pmol of reverse primer, 0.6 U of polymerase (England Biolabs) to amplify all exons except exons 14 and 26 that required 1 U of polymerase (England Biolabs), and 1X PCR Callida's buffer from Callida's Genomics. Exon 14 (3.1 kb) and 26 (1.9 kb) required three and two overlapping nested asymmetric PCR, respectively. All PCR reactions were performed using Gene Amp PCR system 9700. PCR reactions were for 45 cycles with an initial denaturation at 96°C and for 3 min, followed by 96°C for 30 sec, annealing at 55°C for 30 sec and extension at 72°C for 1 min. Final extension was at 72°C for 1 min. An aliquot of the PCR product was analyzed on a 1% (w/v) agarose gel to verify size and quality of the PCR product. The single strands are detected with gel-star Nucleic acid Gel stain (Cambrex, Rockland, UK).

### Probes

Amino-modified unlabeled hexamer (6-mer) probes as well as TAMRA and QUASAR labelled pentamers (5-mer) were manufactured and provided by Callida Genomics. HyChip™ slides contain eight replica arrays of a complete set of 4,096 glass-bound 6-mer probes and hundreds of fluorescent and empty marker dots to guide image analysis. A standard universal set of eight probe pools contains a complete set of 1,024 split in two colors: 512 probes are made with TAMRA and 512 with QUASAR. The pools were prepared by mixing 128 specific TAMRA-dye tagged probes and 128 labelled with QUASAR (Callida Genomics, Sunnyvale, CA) divided in 4 separated pools for one strand and four for the other strand. The emission wavelength of TAMRA is 552 nm and for QUASAR is 670 nm.

### Target preparation

Prior to hybridization, the PCR nested–asymmetric products were precipitated using isopropanol, creating eight different mixes containing single-stranded DNA of the forward and eight containing the reverse strand. The mixes were performed to optimize the analysis results. We created eight different mixes as follow: exons 1 to 5, exons 6 to 9, exons 10 to17, exons 18 to 25, overlapping fragments 14a, 14b, 14c separately and fragments 26a and 26b in the same mix.

Precipitated DNA was resuspended in 40 µ l of dH_2_O and therefore, the double-stranded PCR template was eliminated by restriction digestion leaving single strands intact. The digestion was performed using Callida's pre-made frozen mix in a total volume of 50 µ l with an optimized amount of DNase I (Gibco-BRL, Rockville, MD) at 37°C for 15-20 min followed by inactivation at 95°C for 5 min.

### Hybridization

Single-strand DNA mixes (50 µ l) were adjusted to 73 µ l with dH_2_O and added to a frozen aliquot of Callida's pre-made ligation mix (47 µ l), containing hybridization buffer (New England Biolabs, Beverly, MA) and an optimized amount of T4 DNA ligase (New England Biolabs, Beverly, MA).

The volume of 120 µ l of DNA-ligase mix (27 µ l) was dispensed into each of the eight labelled 5-mer TAMRA and QUASAR linked probe pools pre-aliquoted in a strip of eight small tubes supplied by Callida. Eight probe pools containing target DNA and ligase were mixed thoroughly and 27 µ l was loaded by pipette onto the HyChip cartridge, where it was drawn by capillary forces into the selected hybridization chamber. Hybridization and ligation occurred in a humidity chamber at room temperature for 60 min.

Slides then were hot-washed (60-65°C) to remove non-ligated labelled probes using Callida's detergent (containing washing buffer) for 15 min in an orbital shaker 150 rpm, rinsed four times in dH_2_O and spin-dried at 1500 rpm for 3 min. Slides then were scanned at 20 µ m pixel resolution using a standard array reader GenePix® 4000B setting PMT (532 nm) = 650 and PMT (635 nm) = 750.

### Analysis

The image analysis and base-calling programs are written in C^++^ programming language. The image analysis program takes advantage of an optimized pattern of fluorescent markers and empty spots on the HyChip™ slide to locate the spotting grid. This allows highly accurate automatic grid and spot detection without the need of any user input (such as clicking a corner spot on a display of the tagged image file format [TIFF] image). Robust pattern recognition statistics provide the correct location of spots in imperfect arrays made of shifted subarrays and tolerates a number of missing marker spots. Automated image analysis allows integration of chip scanning with final data analysis. The base-calling software uses an iterative approach to finding the most likely sample sequence, starting from the input reference sequence. For different analyses, it is assumed that the input reference matches the sample sequence more than 90 to 97%. In addition, other limitations (e.g., maximal length of insertions and deletions) are specified. Under these conditions, the algorithm starts by determining the high confidence reference bases, and then testing for various possible mutations to the reference. The program automatically combines hybridization data from two strands or repeated experiments. The following outline lists the main steps of the base-calling process:

1. Normalize/correct raw spot scores for local variation in array preparation and chip loading.
2. Create table of normalized scores for all 4,000,000 11-mers by combining all 128 labelled 5-mer probes from each pool with 4,096 6-mer sequences. Groups of 128 11-mers have the same score.
3. Calculate a base score for each reference position by applying robust statistics on 11 (22 for two strands) overlapping 11-mer intensity scores reading a given base.
4. Use reference sequence segments that have a strong base score (very likely have no mutation) to remove all full match and mismatch signals expected to be contributed by these sequences to the normalized spot scores.
5. Generate all single-base changes to the reference (including insertions and deletions) and calculate mutation base intensity scores (the same statistics used as in step 3) and corresponding *P*-values.
6. Identify candidate mutations with significant *P*-values. Two sequence variants with a high *P*-value would indicate a heterozygote site. For each candidate mutation, modify the reference and calculate the new *P*-values for the region surrounding the potential mutation. Apply a confidence threshold to the new *P*-values and record the accepted mutation.
7. For sequence sites that have low *P*-value for the reference and all single-base variants, multiple mutations (currently including up to 20 base deletions and up to three base insertions), can be tested following the same general scheme (steps 5 and 6). To detect compound heterozygotes, multiple mutation variants have to be searched across the entire sequence length by a special function.

The implemented base-calling algorithm efficiently uses available data for fast determination (few minutes total for both image analyses of two chips and base-calling of a 3-kb sequence) of most likely sequences present in the analyzed sample using common computers (1 GHz processor, 512 MB of RAM). The speed and accuracy is achieved by combining: 1) iterative sequence assembly starting by first finding non-mutated segments; 2) robust base-calling statistics that use carefully normalized hybridization scores of 11–22 overlapping probes; and, 3) a spot signal deconvolution function based on a database of expected hybridization/ligation efficiency factors for each full-match and mismatch 11-mer. An accurate database of probe behavior is established by statistical analysis of sequencing data obtained on 64 kb of specially prepared DNA. This recently introduced signal deconvolution function is critical for accurate analysis of complex multikilobase samples contributing several single mismatch results per each of 32,768 dot scores representing 128 11-mers each.

## Results

**Figure 1:**
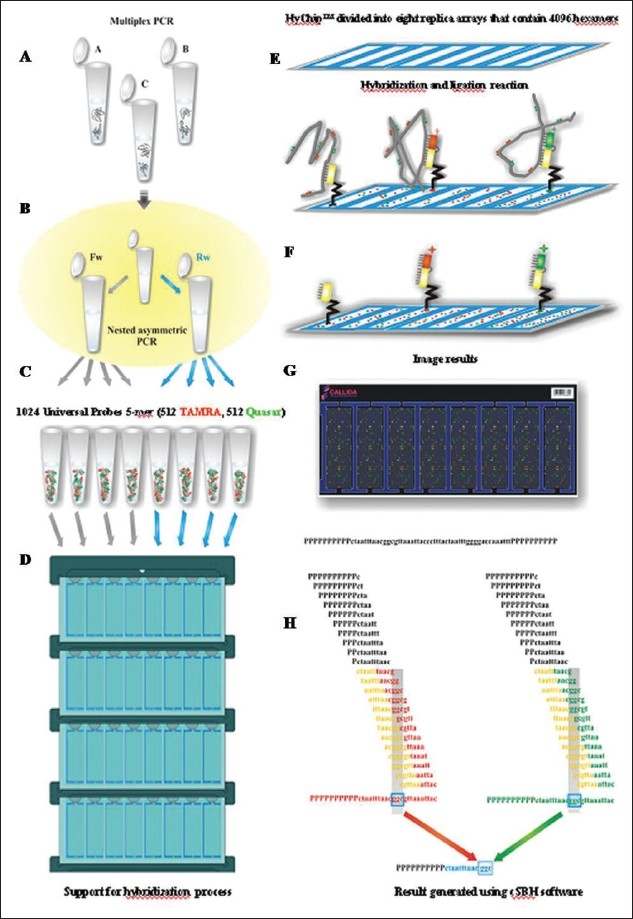
cSBH process. A: Single-stranded nested-PCR template is combined with a ligation mix. B: Mix is dispensed into 8 separate probe pools of 5 mers each containing a complete set of 1,024 split in two colors: 512 TAMRA-labeled and 512 QUASAR-labeled. C: Eight replica arrays of 4096 hexamers each are used to generate a HyChip. A pool mix is loaded onto the array through the loading ports on the slide cover. D: Hybridization at room temperature for 60 min. E: Slides are hot-washed. F: Results are scanned and read on a standard slide scanner. G: Results are scored by image analysis Callida software

**Figure 2:**
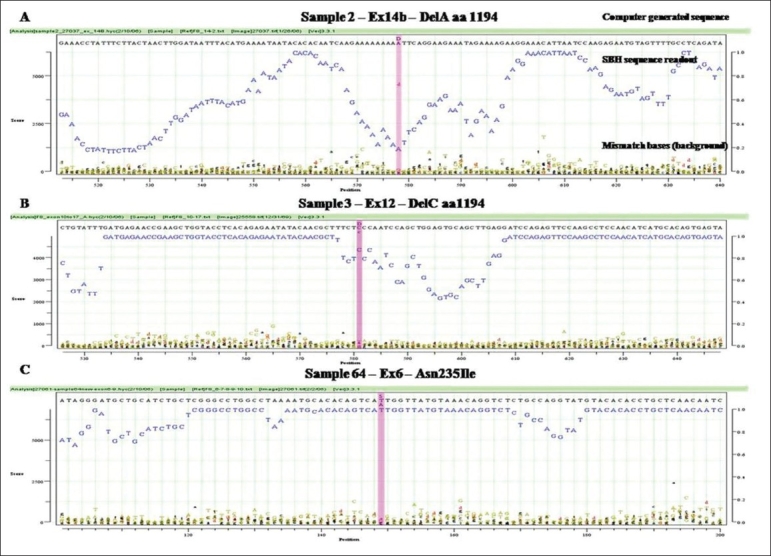
Graphs generated using HyChip™ for three patients. The computer-called sequence is represented on the top line in black; below in blue is the combinatorial sequencing-by-hybridization (cSBH) sequence signal readout on the specific sample tested. At the bottom of the graph are not called by the computer software bases (background). In the panel A the letter D represents an hemizygous deletion of A in Ex12 (sample 2)(DelC aa1194). In the panel B the letter D represents an hemizygous deletion of C in Ex12 (sample 3). In the panel C the letter S represents a T (Sample 64)µ substitution of A

The new 2-color cSBH is an indirect sequencing method in which overlapping probes of known sequence are hybridized to DNA target molecules. In cSBH, which is represented in a schematic overview in Figure 1, target DNA from a PCR product is exposed to two universal sets of short probes in the presence of DNA ligase. When both array-bound and solution-phase labelled probe hybridize to target DNA at precisely adjacent complementary positions, they are covalently linked by DNA ligase, creating one long, array-bound labeled probe of known sequence that is complementary to the solution-phase, single-stranded PCR-generated DNA template. A schematic presentation of a cSBH base-calling chromatogram is shown in Figure 2. The y-axis represents the relative intensity score of the hybridization signal of all overlapping probes at each base. In the presence of strong hybridization signals, the SBH readout appears high, and the reverse is true when there are weak probe hybridization signals. The x-axis represents the position of each base in the sequence analyzed. The computer-called sequence, compared to the known reference sequence used, is represented as the top line of the sequence (in black), and the actual SBH sample sequence readout is directly below (in blue).

Background bases, which are not called by the computer software, are present at the bottom of all graphs. Use of informative pools of labelled probes allows combinatorial ligation of all labelled and bound probes on one glass slide with only eight universal replica arrays of bound probes. This is the most efficient way to obtain hybridization data for all 4,194,304 11-mers using 32,768 (8 X 4,096) probe spots, an array that can fit only one half of all possible 8-mers. Data of pooled 11-mers are much more informative than data for individual 8-mers because each base is read with 11 instead of eight overlapping probes.

## Mutation Detection in Italian Haemophilic A Patients

A total of 16 clinically diagnosed Italian haemophilic A patients and one obligate carrier previously genotyped and found to have disease-causing mutations in the FVIII gene were included in this study. Patient samples included a range of DNA mutations mapping throughout the FVIII gene. cSBH results are shown in Table 2. We confirmed single-base changes in samples 1, 20, 64, 65, 80, 88, 90, 93, 99. Previous genotyping of samples 2 [Figure 2A], 3 [Figure 2B], 37, and 89 carrying a single-base deletion in exon 14 (fragment b) as well as in sample 6 [Figure 2C] containing a single deletion in exon 12 was confirmed by cSBH. In addition, we found polymorphism in the 3’UTR region (Ex 26, nucleotide 8900 (G→A)) in sample 2, 3, 37, 73, and another polymorphism in the 3’UTR region at nucleotide 8519 A→G in sample 3.

**Table 2:** HA cSBH screen results in 16 patients with known and in 7 patients with unknown mutations

Sample	Sequencing results	cSBH results	
		
	Sporadic mutations	Sporadic mutations	Gene polymorphism
# 1	Lys1040Stop	Confirmed	
# 2	Del A Aa 1191-4	Confirmed	UTR 3’ G→A 8900
# 3	Del C Aa 596 novel	Confirmed	UTR 3’; A→G 8519
			UTR 3’ G→A 8900
# 6	Del A Aa 1191-1194	Confirmed	
# 20	Lys1270Stop	Confirmed	
# 37	Del A Aa 1191-1194	Confirmed	UTR 3’ G→A 8900
# 64	Asn235Ile novel	Confirmed	
# 65	Asn235Ile novel	Confirmed	
# 73▵	DelTT Aa 2275	Confirmed*	UTR 3’ G→A 8900
# 80	Arg1997Trp	Confirmed	
# 88	His94Gln	Confirmed	
# 89	Del A Aa 1441	Confirmed	
# 90	Pro74Ser novel	Confirmed	
# 93	Arg1997Trp	Confirmed	
# 95	Del CT Aa 49 novel	Confirmed*	
# 99	Arg1997Trp	Confirmed	
# Cz36	unknown	unknown	UTR 3’ A→G 8519
			UTR 3’ G→A 8900
# P21	unknown	unknown	UTR 3’ G→A 8900
# P25	unknown	Leu1843Thr	
# R32	unknown	unknown	Ser 2182 His
# Cz1	unknown	unknown	UTR 3’ A→G 8519
			UTR 3’ G→A 8900
# Cz13	unknown	unknown	UTR 3’ G→A 8900
# Cz34	unknown	unknown	UTR 3’ G→A 8900

* Genotyping was accomplished using a more advanced version of the software

The correct genotyping of a patient (sample 95) and an obligate carrier (sample 73) with 2-bp deletions starting at codons 2275 and 49, respectively, was accomplished using a more advanced version of the software.

We also analyzed samples of 7 haemophilic patients with unknown mutations, who were excluded to carry one of the common HA mutations, inversion of the intron 22 or intron 1. In this setting, we have found a previously undetected missense mutation Leu1843Thr in exon 16 in the sample P25. The mutation was then confirmed by direct gene sequencing. In sample R32 we found another polymorphic change in Ex 23 (Ser 2182 His). In addition, we found a polymorphism in the 3’UTR region (Ex 26, nucleotide 8900 (G→A)) in sample P21, Cz1,Cz13 and Cz34 and Cz36 and another polymorphism in the 3’UTR region at nucleotide 8519 A→G (samples Cz 36 and Cz 1).

## Discussion

We demonstrated that cSBH, and the related HyChip product, is capable of identifying a range of point mutations located within the FVIII gene making this platform an attractive alternative to standard sequencing methods.

We confirmed known FVIII gene mutations and found that large regions of genomic DNA can be sequenced with excellent readability using only one HyChip array. Because the maximal length of a single PCR product that could be sequenced by the chip is 1.2 kb in size, long exons such as exons 14 and 26 had to be divided in three and two fragments, respectively. The other exons are shorter and could be sequenced in entirety. cSBH sequence data showed 100% accuracy with only 0.2% of bases not called.[10] In addition, we demonstrated that the smaller exons 1-25 could be pooled together in groups in a manner allowing their complete sequencing on four different chips, with base readability of 100% and an accuracy of 100%.

The reliability of this technique has been further provided by analyzing a series of HA patients in whom no gene mutation was detected by the first FVIII gene screening, using common strategies (SSCP, DGGE, or dHPLC). This approach was able to identify a new point mutation (Leu1843Thr), which was subsequently confirmed by direct gene sequencing. In the remaining patients, no mutation was found also when the direct gene sequencing approach was applied. Finally, how well this technique does work was provided by the identification of the mutant allele in a heterozygous carrier with one normal factor VIII gene.

Recently, a different approach using a microarray technology has been applied for the detection of FVIII gene mutations.[14] As compared with that, the method we used was able to screen all exons using universal probes, rendering this approach reliable for all HA patients carrying a point mutation wherever it is located and chips available for a screening of different HA patients or distinct genes.

cSBH is an efficient, reliable, and easy to use re-sequencing technique that offers an alternative approach for screening of large genes. cSBH allows sequencing of large segments of DNA on only one chip, thereby drastically reducing the time it takes to analyze a specific sequence. Actually the mean time to screen the coding regions of FVIII gene was three days. This period was reduced as compared to the time used in standard methods and comparable to that needed for sequencing analysis. The only equipment required, aside from basic tools (e.g., thermal cyclers, pipettes), is access to a standard array scanner, which makes it a very affordable technique to implement. The required data analysis software, which runs on standard PC computers, will be provided as part of the universal HyChip system and is expected to be economically priced. cSBH is a non-radioactive method and generates sequencing results that can be obtained from genomic DNA within a day. Another major advantage of cSBH are the truly universal HyChip arrays which can be applied to screen any DNA sequence without further probe or oligonucleotide changes, which can be very costly. cSBH offers an efficient and accurate screen for most DNA sequences. The cSBH method is highly reproducible because each base is currently read with 22 overlapped probes (11 per strand). In addition, sample preparation and ligation kits, aliquoted for individual assays, are prepared with all components including enzymes to yield very reproducible biochemical reactions. Furthermore, the base-calling software includes several normalization functions to minimize the influence of experimental variations.[13]

The combinatorial ligation of universal probe sets coupled with informative probe pools provides a large number of long (currently 11-mers, potentially 12- to 15-mers in two-probe ligation and 17- to 24-mers in three probe ligation), overlapping probes of predetermined behavior that interrogate each base. Robust statistics take advantage of this redundant dataset to provide accurate base calling in spite of array imperfections (0.5–5.0% of missing dots; high droplet size variation), some impaired probe-target hybridizations due to target–target hybridization, significant experimental noise, and the always-present statistical noise related to probe-pooling and sequence repeats. The main software improvement in the future should address target DNA behavior (e.g., primers/primer dimers, palindromes, direct and inverted repeats, and uneven amounts of target DNA) to aid in a more accurate interpretation of base scores and associated *P*-values. Actually, the presence of short palindromes or repeats may contribute to the number of bases with lower “scores”, as seen in sample 2 [Figure 2A]. Furthermore, detection of low-signal AT rich sequences can be improved with optimization of probe concentrations and incorporation of 1,600 AT-rich 7-mers and 8-mers in the arrays, as well as 500 AT-rich 6-mers and 7-mers in the labelled probe pools, which are currently under development.

Universal arrays use probes shorter than gene specific arrays. As a consequence, there is a higher chance to have repeated sequences longer than the length of probes (currently 11-mers in cSBH) occurring in a DNA sample. Such sequences may lead to uncertainty in the sequence assembly. For example, a stretch of 12 As can be perceived as 11, 12, 13, or more As. Also, a mutation in the middle of a repeated 20-mer or any longer segment may not be assigned to the actual copy of the repeat. This situation can be avoided by amplicon design that separates repeats into two sequencing reactions. Short identical repeats several bases in length or longer imperfect repeats, both found in exons or immediate intronic sequences that flank each exon, usually do not confuse the advanced base-calling software.

There are limitations with cSBH analysis: the current software and probe set must be improved to detect DNA alterations within simple repeat regions, which is a problem common to other sequence technologies as well.[10]

Another potential complication, which is common to other high resolution methods, including standard Sanger sequencing, is that all sequence changes, polymorphisms and missense changes that do not result in an obvious pathogenic mutation such as a stop codon and are of unknown clinical significance will be detected.[10]

In summary, mutation detection for the FVIII gene requires a multimodal approach that can detect the full range of mutations present in this gene. First-stage mutation screening requires a fast, efficient and possibly economical approach to scan the entire gene. cSBH is a technique that is ideally suited. In this study, we showed that cSBH is capable of detecting a broad range of mutations, currently has the capacity to analyze long continuous read lengths of up to 2 kb per chip, and is able to analyze pooled PCR fragments (such as exons 1–25 in this study) on a single universal chip.
